# A Panorama of Extracellular Vesicle Applications: From Biomarker Detection to Therapeutics

**DOI:** 10.1021/acsnano.4c00666

**Published:** 2024-03-12

**Authors:** Sumita Das, Christopher J. Lyon, Tony Hu

**Affiliations:** Center for Cellular and Molecular Diagnostics and Department of Biochemistry and Molecular Biology, Tulane University School of Medicine, New Orleans, Louisiana 70112, United States

**Keywords:** extracellular vesicles, techniques, detection, analysis, disease biomarkers, diagnostic, therapeutics, nanocarriers, targeted delivery

## Abstract

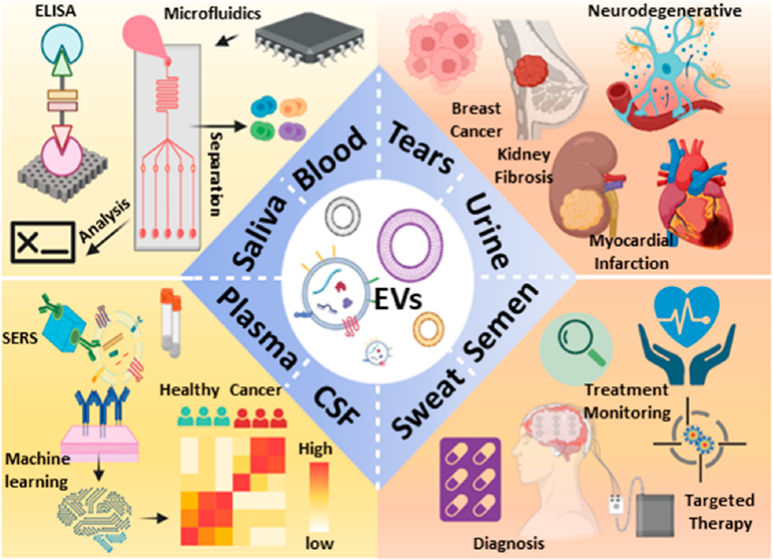

Extracellular vesicles (EVs) secreted by all cell types are involved in the cell-to-cell transfer of regulatory factors that influence cell and tissue phenotypes in normal and diseased tissues. EVs are thus a rich source of biomarker targets for assays that analyze blood and urinary EVs for disease diagnosis. Sensitive biomarker detection in EVs derived from specific cell populations is a key major hurdle when analyzing complex biological samples, but innovative approaches surveyed in this Perspective can streamline EV isolation and enhance the sensitivity of EV detection procedures required for clinical application of EV-based diagnostics and therapeutics, including nanotechnology and microfluidics, to achieve EV characterizations. Finally, this Perspective also outlines opportunities and challenges remaining for clinical translation of EV-based assays.

## Introduction

Extracellular vesicles (EVs) play essential roles in cell communication and regulation, including several processes associated with disease pathology since these nanoscale particles are abundantly secreted by all cell types to induce local and systemic effects, particularly during cell injury responses. Significant effort has focused on developing methods for rapid, accurate, and sensitive detection of specific EV populations associated with disease processes through targeting EV-specific biomarkers because EVs carry a wealth of information and are present at high abundance in most of the body fluids to simplify the collection of diagnostic specimens. These methods target arrays of biomolecules (proteins, mRNAs (mRNAs), microRNAs (miRNAs), long noncoding RNAs (lncRNAs), DNAs, lipids, and metabolites) expressed by EVs secreted from injured or diseased cells as disease biomarkers. Such EV-derived biomarkers contain rich diagnostic and prognostic information, as they can regulate intracellular communication, cell maintenance, cell maturation, and innate and adaptive immune responses associated with disease development and pathology. EV-derived biomarkers have thus been proposed as candidate biomarkers for several infectious chronic and malignant disease conditions.^1^ However, sensitive and accurate detection of specific EV biomarkers is complicated by the heterogeneity of EV populations that are present in biological specimens and by the presence of various nanoparticles (protein aggregates, lipoproteins, cell debris, etc.) that can contaminant bulk EV preparations. Single EV analysis methods have been proposed as a means to avoid this problem and evaluate the full spectrum of EVs and their potential roles in disease development and pathology. Such single EV analysis methods are still in their infancy, however, due to incomplete understanding of EV biogenesis, technical limitations, and the current lack of standardization and validation approaches for these analyses. Substantial research has also been performed to develop EV-based therapeutics that exploit their highly desirable properties (robust *in vivo* stability, tissue permeability, cell/tissue selectivity, and drug loading capacity) to permit the targeted regulation of processes associated with disease development and progression in specific cell types. This includes the use of various methods (genetic engineering, transfection/fusion, chemical modification, etc.) to modify the content or surface molecules of distinct EV populations and thus alter their regulatory function or cell targeting specificity. However, challenges for clinical EV diagnostic applications range from difficulties in the collection, purification, quantification, and handling of EV samples; targeting EVs derived from specific cell types; and poor understanding of their pathological effects. Minimal Information for Studies of Extracellular Vesicles (MISEV) guidelines, which include recommended protocols for EV characterization, handling, and analysis have made the process for developing EV therapeutics intended for use in clinical settings more straightforward.^2^ Similar method-specific guidelines have proven to be useful in facilitating the development of diagnostics that employ quantitative real-time PCR, flow cytometry, and other specialized methods that require standardized guidelines to produce reliable data. Use of automated systems for EV isolation and characterization has also accelerated the development of clinically feasible EV-based diagnostic applications.^3−5^ Standardization of EV handling procedures still needs to be developed to ensure that biomarker integrity is preserved prior to analysis, however, particularly for samples intended for early stage diagnosis where signal intensities may be very low or when evaluating longitudinal samples for increase in low concentration EV biomarkers associated with disease development or progression. Standardized methods of EV preparation and characterization approaches are also required to ensure that the EV therapeutics exhibit consistent safety and efficacy. Further research is necessary to identify EV biomarkers associated with effective treatment responses, although “omics” studies can provide substantial insight on this topic. Clinical applications using EV diagnostics or therapeutics will require rational approaches that streamline workflows and improve the performance of current methods. We therefore describe the advances and remaining challenges for EV-based diagnostics and therapeutics.

## Advances in EV Detection Techniques

Conventional EV analysis methods, including polymerase chain reaction (PCR), Western blotting (WB), and enzyme linked immunosorbent assay (ELISA) are expensive and time-consuming, can have moderate sensitivity, and usually require a separate EV purification step, all of which can reduce their ability to be incorporated into clinical applications. However, recently great progress has been made in refining EV analysis methods to enhance their sensitivity, specificity, and practicality for use in clinical settings, and tailor-made nanomaterials and microfluidic platforms are being explored to further improve their performance characteristics. Fluorescence-, surface plasmon resonance (SPR)-, surface-enhanced Raman spectroscopy (SERS)-, electrochemical-, and aptamer-based EV detection approaches coupled can all be employed to permit sensitive signal recognition in EV detection platforms.^6^

### Nanomaterial-Based Approaches for Sensitive EV Assays

**Figure 1:**
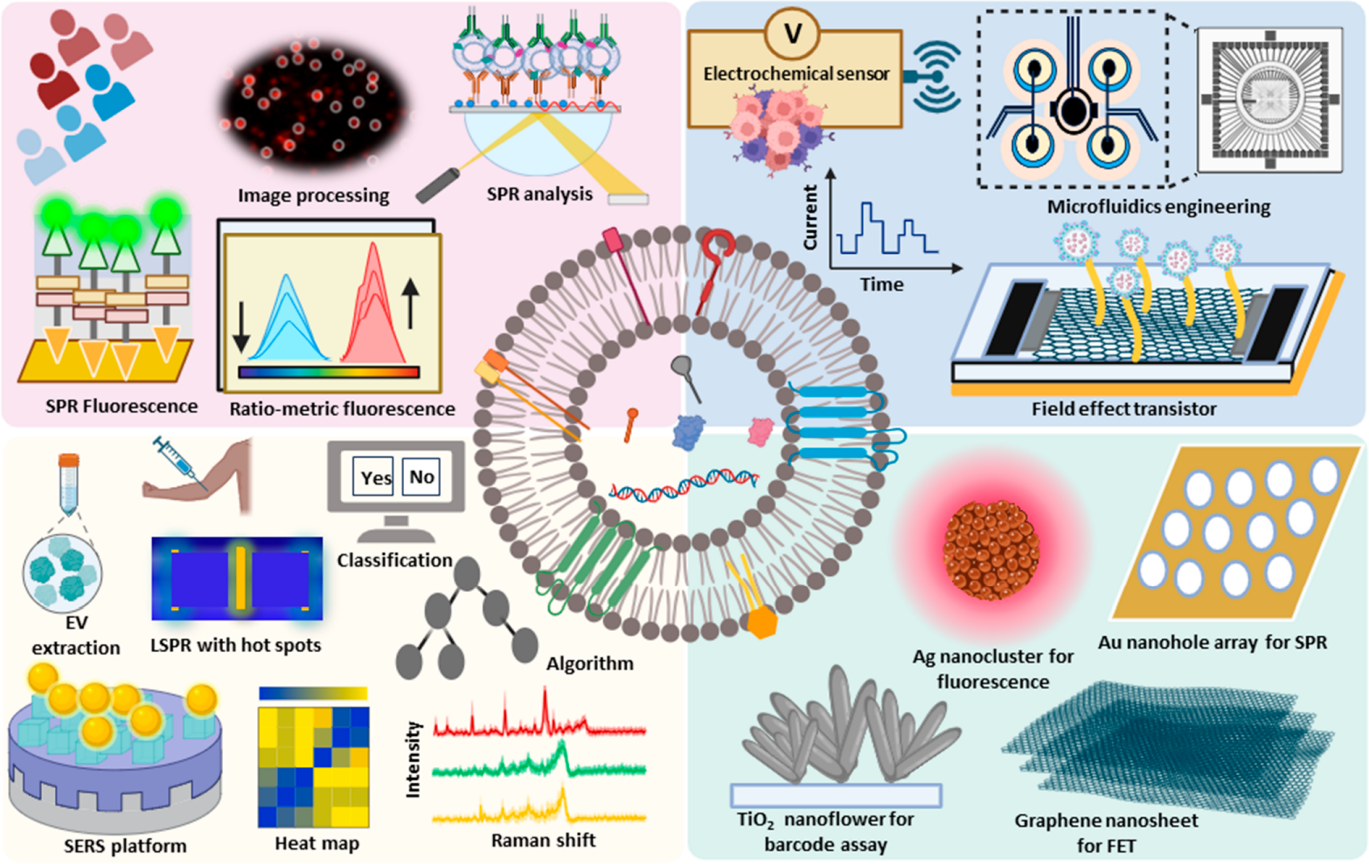
Schematic depicting various EV detection technologies employing fluorescence (upper-left), surface plasmon resonance (SPR) (upper-left), surface-enhanced Raman spectroscopy (SERS) (lower-left), electrochemical and microfluidic approaches (upper-right), and the nanostructures used for EV detection methodologies (lower-right). Figure created with Biorender.com.

Nanomaterial research has led to the discovery of nanoscale material properties that can be tuned to enhance the sensitivity or specificity of EV assays or streamline their workflows to render them suitable for use in clinical applications. Recent research has focused on applying nanomaterials with fluorescent, SPR, SERS, and electrochemical approaches to detect low concentration target EV subpopulations, including those in complex samples (Figure 1). Such approaches require use of nanomaterials with appropriate physicochemical properties to achieve ultrasensitive EV detections. Standard assays that employ fluorescent reporters to detect specific biomarker targets can have high sensitivity and specificity and provide multiplex readouts, but high autofluorescence background signals frequently detected in complex clinical specimens can be a major concern for assay sensitivity. The use of fluorescent or fluorescently tagged nanomaterial reporters, or nanomaterials with other optical properties, can partially alleviate some of these issues. For example, the optical properties of nanomaterials are influenced by their size and chemical composition, and thus it is possible to design materials that have high signal intensities within a narrow range that can be adjusted to avoid fluorescent background from the specimen. Nanomaterial-derived optical signals are also not subject to common photobleaching effects that can rapidly degrade fluorescent signals detected from organic dyes, and fluorescent nanoparticles have been used to replace organic dyes used to label affinity probes to take advantage of these properties for EV analysis assays.

DNA nanomaterials have also been used to simplify the synthesis and labeling of affinity probes and reduce their cost since aptamers specific to a biomarker target can be rapidly and cheaply synthesized after their initial identification and labeled with high efficiency at defined positions either during or after their synthesis. For example, one recent study employed magnetic nanoparticle conjugated with a DNA machine to detect target EVs.^7^ In this approach, binding of an aptamer to an EV target protein produced a conformational change that induced a rolling circle amplification reaction to amplify a G-quadruplex region bound by a reporter dye and the loss of a silver nanocluster-labeled reporter oligonucleotide, with target binding analyzed by the change in the ratio of these two signals.

SPR signals generated by changes in the refractive index of a plasmonic surface due to the molecular interaction events have been used as a label-free means for EV analysis. SPR assays employed for EV analysis have increasingly used nanomaterials since the specific properties of these materials can increase SPR signal characteristics to enhance EV detection sensitivity. For example, a SPR design employing the signal generated by dual-binding of gold nanosphere and nanorod biomarker probes to EVs captured by EV-specific antibodies was found to markedly increase the detection sensitivity of the plasmon sensor to allow the detection of stage-I and stage-II pancreatic cancer patients.^8^ Recent SPR assay approaches used for EV analyses have employed several distinct types of nanostructures, including nanoellipsoids, nanoshells, and an SPR sensor containing a periodic array of nanoholes, which had specific advantages and disadvantages.^9^ For example, the SPR assay that used a periodic nanohole array had higher sensitivity than conventional SPR sensors due to its tunable optical resonance properties, which could be adjusted by altering the parameters of its nanohole array, although this sensitivity was also influenced by the rate of EV diffusion to the sensor surface to permit the capture of specific EV subtypes. To address this limitation, electroosmosis and dielectrophoresis forces were applied to an advanced plasmonic EV platform to rare cancer EVs in plasma.^10^

SERS signal is produced by the inelastic scattering of discrete units of quantum or vibrational energy (phonons) from a thin metal surface and can be altered by changes produced in the electromagnetic field of an affinity factor-conjugated surface when it interacts with its specific biomarker target. SERS sensors have been used to analyze the composition of target EV populations and have the potential to facilitate the rapid analysis of large numbers of sample. Further, the performance of SERS-based strategies for EV detection can be improved by using nanostructured SERS substrates designed to optimize the size and morphology of the sensor substrate. By example, a recent study employed a nanomaterial SERS sensor to increase the signal detected upon target EV binding in order to allow ultrasensitive detection of circulating cancer-derived EVs to diagnose glioblastoma.^11^ Signal enhancement observed with this sensor were attributed to quantum effects produced by the 3D cubical architecture of its Ni nanostructures and their efficient charge transfer mechanism. However, the steric hindrance effects resulting from its 3D architecture could attenuate EV adhesion to limit the overall EV binding capacity and signal production. Functionalization of this 3D Ni architecture with a Au/Pd network was subsequently found to enhance its SERS signal production 3.6-fold through this secondary surface anchoring network.^12^ Similarly, another study found that the surface state of a semiconductor TiO_2_ nanostructure sensor was a major factor in determining the SERS signal produced upon capture of cancer stem cell-derived EVs.^13^ Specifically, nanosensors with the highest degree of oxygen vacancy were found to boost SERS signal ∼1000-fold versus those with a low degree of oxygen vacancy.

Electrochemical-based EV assays that employ nanomaterial substrates are also useful for the rapid, sensitive, inexpensive, and label-free detection of target EV populations. Such approaches can also be easily combined with miniaturized and automated electronic devices desirable for use in point-of-care applications or resource-limited clinical settings. These assays typically capture target EVs using specific antibodies or aptamers conjugated to an electroactive transducer and measure the signal these capture events produce via amperometry, voltammetry or field-effect-transistor based signal readouts. A single potentiostat can frequently be used to analyze signals produced by multiple electrochemical cells to enhance assay throughput and reduce equipment costs. For example, one such nanoparticle-based immunoassay approach employed a 16-well electrode microplate design where wells were connected to counter/reference electrodes to quantify biomarker levels present on urinary EVs (UEVs).^14^ Similarly, another study used a 96-well electrochemical assay platform to analyze signals produced by antibody coated magnetic beads that were specific for selected EV biomarker targets.^15^ A field-effect transistor (FET) electrochemical assay employing an integrated antibody-conjugated 2D graphene functionalized gate electrode (carrier mobility >2000 cm^2^ V s^–1^) has also been used to detect a biomarker-positive EV population in plasma samples.^16^ This approach employs high quality graphene that is grown by chemical vapor deposition to generate sensitive and inexpensive sensors that can be produced at scale.

However, while most EV isolation methods use affinity molecules to capture target EVs from biospecimens, the physical properties of nanomaterials can sometimes also be used to capture bulk EVs. For example, one group employed the topology of TiO_2_ nanoflowers to enhance their interaction with EV surface phospholipids to promote their capture as this nanoflower structure allowed individual EVs to fit between and form close interactions with several spikes of a nanoflower.^17^ EVs captured on these nanoflowers were then analyzed by using a barcode approach to detect procoagulant factors associated with venous thromboembolism that can be induced by tumor-secreted EVs in cancer patients. Similarly, another group has used the high positive charge of a ZnO core/Al_2_O_3_ shell nanowire substrate for EV capture to increase EV concentrations on this sensor to increase assay sensitivity for targeted EV membrane proteins.^18^

There is a growing interest in the use of aptamer-based methods for EV assay platforms since these single-stranded nucleotide structures can undergo conformational changes upon interaction with a biomarker to induce nucleic acid amplification cascades or signaling amplification reactions. Such conformational changes can expose a target sequence to allow its amplification or a previously repressed RNA- or DNA-based enzyme activity. This direct coupling of target recognition to signal production can be highly advantageous, particularly due to the ease and low cost of producing aptamer sequences at scale. Further, while aptamers have low affinity versus comparable antibodies, this is offset by the difficulty and expense of tagging such antibodies with nucleic acid reporters to allow comparable signal transduction upon target recognition.

Nanomaterials can provide several advantages for EV assays, but EV assays that use this approach can still be subject to issues that can limit their usefulness for clinical applications, such as the potential photobleaching or autofluorescence effects that can limit their sensitivity. However, such limitations can often be attenuated by using additional detection or analysis approaches. For example, nanostructured SPR sensors, which have good resolution and permit multiplex detection of distinct EV targets, can be employed in fluorescence-based detection strategies to permit more sensitive EV detection. Similarly, applying machine learning algorithms to SERS-based detection approaches can also substantially improve the detection sensitivity of these assays. Despite the theoretical promise of these EV detection approaches, none of them have yet been adapted for routine use in clinical settings.

### Microfluidic EV Detection Methods

Microfluidic devices represent promising benchtop approaches for EV detection since they permit multiple sample processing steps to be incorporated into a single assay platform to permit automated high-throughput analysis of small volume diagnostic samples. Several microfluidic EV isolation platforms have now been developed that employ various signal amplification and detection strategies to detect target EV biomarkers in their isolated EV samples. Minor differences in microfluidic assay platforms can have substantial effects on the performance of these devices for EV capture and analysis since nanoscale EV particles are highly susceptible to changes in shear forces and turbulence related to channel dimensions, geometry, and flow rates, and these effects can be employed to control EV interactions with the microfluidic device and assay reagents. Serpentine channels, microstructures, or arrays of nanoscale pillars can, for example, be used to promote turbulence and homogeneous mixing of sample EVs and assay reagents or EV dispersion over a capture or analysis surface. Surface functionalization of the channels and capture analysis surfaces of these devices can also play important roles in minimizing the nonspecific interactions and promoting affinity capture reactions required to reduce background and increase EV capture for sensitive detection.

Lithography and 3D printing are the most frequent methods used to fabricate microfluidic devices that can be coupled with signal amplification and detection strategies to permit rapid and sensitive EV detection, and several distinct designs have been used for this purpose, although SERS detectors are commonly used in these designs. For example, one recent study used a 3D printed polydimethylsiloxane (PDMS) microfluidic chip containing embedded arrays of plasmonic nanocavities size to contain single EVs to achieve single EV resolution when read by a label-free method that detected SERS signals associated with EVs derived from different cell types.^19^ A second group used a SERS-based microchip design in which a specific antibody was used to directly capture target serum EVs, which were then hybridized with a set of SERS nanotags to allowed multiplex analysis to detect early melanoma, using sensors that contained asymmetric circle-ring electrodes that also promoted nanoscopic flows to enhance sample mixing.^20^ A third group used an alternate design where antibody-conjugated nanoparticles were used to magnetically sort EVs into different populations based on their relative expression (negative, low, medium, and high) of selected membrane biomarkers and to allow the subsequent analysis of the distinct EV isolate populations.^21^ A fourth group employed a paper-based SERS-vertical flow biosensor for multiplex EV detection and found that this approach reduced cross-reactivity and false negative results.^22^

However, while multiple groups have employed SERS for their detector readouts, a few groups have used alternate approaches. For example, one group coupled a microfluidic structure containing three independent flow channels with an antibody-functionalized patterned dielectric metasurface that enhances the local electric field to produce strong signal upon interaction with target analytes to allow multiplex EV analysis, which they proposed as a means to allow rapid detection of breast cancer derived EVs without sacrificing analytical performance or reproducibility.^23^

Microfluidic platforms demonstrate significant promise for the development of EV-based assays suitable for use in clinical applications (Table 1), but practical considerations such as their complicated and time-consuming fabrication processes have limited their production at a scale, and available EV platforms have not been subjected to necessary clinical validation studies.

**Table 1:** Current Microfluidic Platforms Have Been Developed for EV Detection

device design	source	detection platform	application	multiplexing	ref
3D printing	Plasma	SERS	Glioblastoma	Yes	(19)
Photolithography	Serum	SERS	Melanoma	Yes	(20)
Photolithography	Plasma	Nanoscale cytometry	PD-L1 expression	No	(21)
Paper based	Serum	SERS	Breast cancer	Yes	(22)
Lithography	Plasma	Opto-fluidics	Breast cancer	Yes	(23)

## Single EV Analysis Techniques and Their Diagnostic Potential

**Figure 2:**
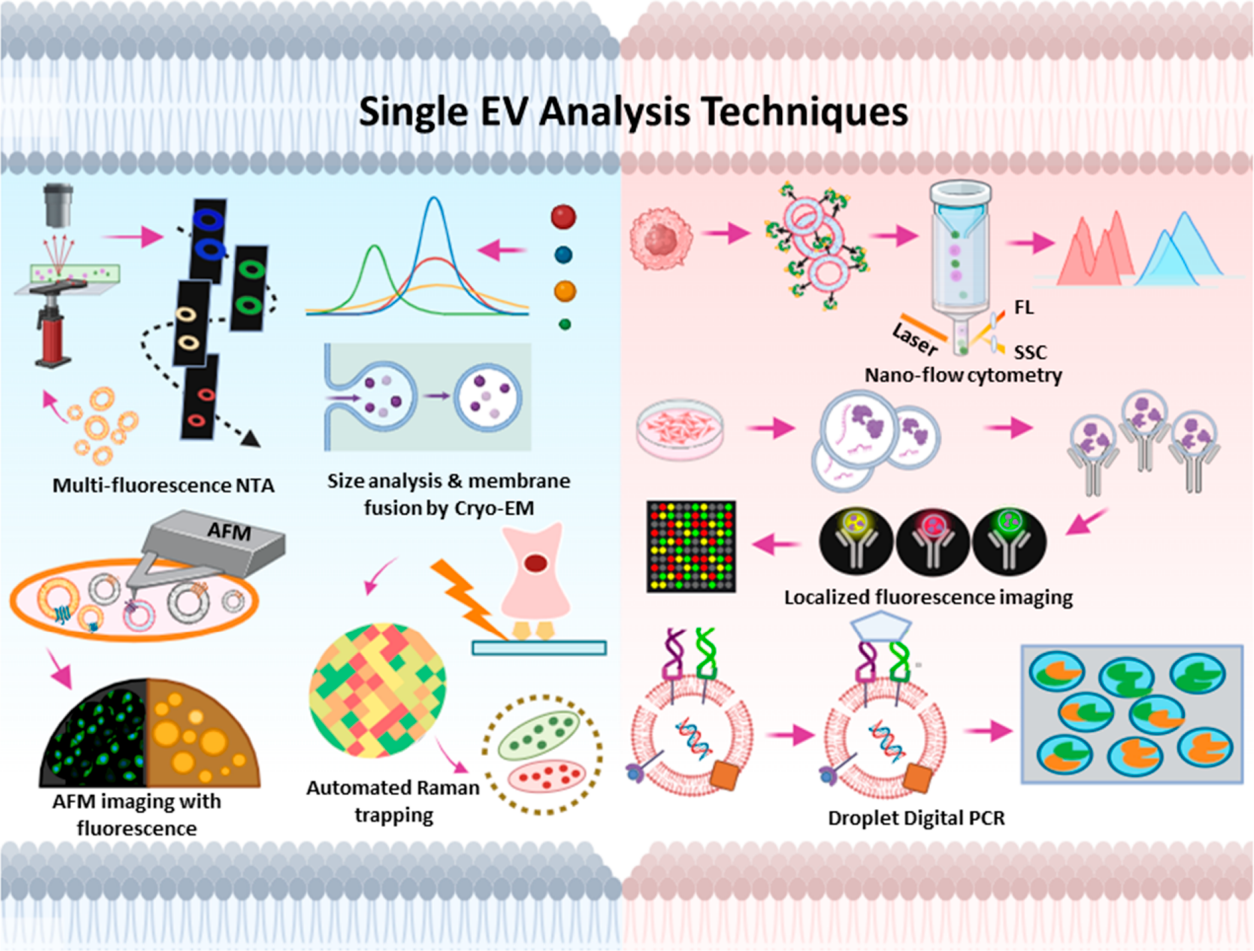
Schematic of current single EV analysis approaches using label-based and label-free detection methods. Figure created with Biorender.com.

Development of advanced methods to allow biophysical characterization at single EV resolution has been a major focus for recent translational EV research studies. EVs present in biospecimens are highly heterogeneous since they may contain factors reflecting the phenotype of various cell types in distinct tissues (e.g., plasma EVs), cells with different lineages or fates states within a tissue, or cells with different genetic backgrounds within a tumor. Single EV analysis techniques can assess aspects of heterogeneity of EV subpopulations (e.g., size and composition), detect EV biomarkers present at a low concentration in complex specimens, and detect the association of specific EV biomarkers on single EVs or EV subpopulations, all of which could provide information about disease development and progression or treatment responses that would now be detectable in EV analysis performed across complex samples. Single EV analysis approaches employ a variety of techniques, several of which do not require the use of exogeneous labels (Figure 2), including nanoparticle tracking analysis (NTA), cryo-electron microscopy (cryo-EM), atomic force microscopy (AFM), and laser-tweezer Raman spectroscopy approaches.^24^ However, many of these approaches provide limited information when used in a label-free manner or are not useful without independent EV isolation procedures. Label-free approaches that employ NTA can only provide estimates of the distribution of EV diameters in a population, while cryo-EM and AFM methods can also provide some information about the distribution of their conformational phenotypes, and all three methods can have difficulty discriminating EVs from protein aggregates and viruses that may contaminate EV isolate fractions. Single EV analysis methods have thus increasingly employed fluorescent affinity tags to detect specific EV subpopulations for analysis, including one study that employed four laser wavelengths to distinguish distinct EV subpopulations in a multiplex NTA analysis assay.^25^

Similarly, multiparametric AFM imaging has also been combined with high-resolution fluorescence microscopy to analyze differences in the molecular, morphological, and mechanical characteristics of EVs derived from different cell populations. Notably, one study found that a similar multiparametric AFM imaging approach detected differences in the mechanical properties (e.g., Young’s modulus) and viscosity of EVs present in the blood of patients with hematologic cancers that could distinguish them from those in the blood of healthy patients.^26^

Nonetheless, label-free methods can still provide useful information about the overall EV population or distinct EV subpopulations isolated by their individual properties. Cryo-EM is considered a gold standard approach for direct observation of EV morphology, since it avoids potential staining, fixation, and dehydration effects associated with other EV analysis methods. This approach has provided important information about the internal structure of individual vesicles, intermediate stages in the membrane fusion processes that regulate EV cargo delivery, and EV structure under different conditions.^27^

Raman spectroscopy analyses can provide information that can distinguish individual EV populations but require approaches that allow rapid processing of significant numbers of EV to be effective. Single particle automated Raman trapping analysis (SPARTA) can automatically capture single EVs in a laser focal point to record their Raman spectra and then analyze differences in these spectra using a dimensional reduction analysis approach that allows rapid characterization and high-throughput EV comparisons (>100 measurements per sample).^28^ This approach was used to conduct a comprehensive analysis of EVs derived from cancerous and noncancerous breast tissue cell lines and found to distinguish EVs from cells belonging to these two categories with high sensitivity and specificity (>95%), although this approach has not been replicated with EVs from human blood sample or biopsies.

After years of effort, flow cytometry analysis (FCA) has also been adapted to permit its use with nanoscale EV targets and has the potential to play a prominent role in EV subpopulation analyses due to its high-throughput and multidimensional capabilities and ability to use widely available fluorescently tagged antibodies or aptamers. High signal intensities required for such analyses have been achieved by optimizing flow rates inside a microfluidic channel to obtain a high (10 kHz) read rate when using an alternative cross-correlation analysis strategy to ensure robust multicolor colocalization with single-vesicle reads.^29^ Refractive indexes of individual EVs can also be estimated at this read rate by combining side-scattered light detection results with Mie theory calculations.^30^ Further, integrating super resolution imaging into nano-FCA approaches can also be employed to characterize EV structure.^31^

Fluorescence-based microscopy image analyses have also been employed as a more straightforward means for EV analysis, since images collected by laser scanning confocal microscopy can have low background signal and minimal interference from out-of-focus objects. One group exploited this capacity to detect a fluorescent signal produced by primer exchange reactions initiated by recognition of a target miRNA (miR-21) after EVs expressing programmed death ligand 1 (PD-L1) were affinity-captured directly from plasma samples on an assay chip and induced to fuse with reagent-loaded nanoscale liposomes.^32^ This simple, high-throughput approach distinguished plasma samples from breast cancer patients from those of healthy donors or patients with benign tumors with 80% sensitivity and 90% specificity. Sensitive single molecule imaging techniques, such as total internal fluorescence microscopy (TIRFM), can also provide enhanced signal-to-noise ratios and localization data to improve the identification and quantification of cancer-specific mutant EV proteins. For example, one study used this approach to identify a significant difference in the expression of the KRAS^t^ and P53 mutant biomarkers in single EV for the diagnosis of stage I and II pancreatic ductal adenocarcinoma (PDAC).^33^ SPR microscopy has also been used to permit high-throughput analysis of single EV analysis of target surface makers by measuring the kinetics of EV interaction with an affinity-labeled surface as specific and nonspecific EV interactions with this surface exhibit different interaction intervals to allow high sensitivity detection of target EVs against a 350-fold excess of nonspecific plasma EVs.^34^

### Digital EV Analysis Approaches

It is often difficult to detect low concentration EV biomarkers of targeted EV subsets, particularly when they are analyzed against the background of the total EV population of a complex biological specimen. However, digital detection approaches that employ nanoliter reactions and can be adapted to lab-on-chip platforms are increasingly used to provide sensitive, reproducible, and quantitative detection of these factors.^35^ These approaches require the segregation of an EV sample into a large array of nanoliter volume reactions that contain one or fewer of the target EVs. Each reaction produces a single positive or negative signal following a signal amplification reaction to indicate the presence or absence of an EV target and allow their absolute quantification in a sample via Poisson-based statistical analysis. Further, digital droplet-based microfluidic assays can also be used for multiplexed profiling of EV target proteins. One such study hybridized EV samples with antibodies conjugated with target-specific DNA barcode tags and captured antibody-modified EVs and removed unbound antibody, by hybridizing these samples with magnetic beads tagged with DNA barcode tags containing a sequence complementary to one present on all the barcode tags. These beads were then encapsulated in digital droplets after adjusting the flow rate, input bead concentration, and droplet volume to yield a Poisson distribution. Bead tags were then polymerase-extended, transcribed into RNA, and RT-PCR amplified to generate an amplicon sequencing library to quantify the abundance of the targeted EV proteins.^36^ Digital approaches for single EV analyses (Table 2) have thus far focused on early cancer detection and evaluation of cancer responses to treatment, although this approach holds promise for other disease applications.

**Table 2:** Various Recent Single EV Analysis Techniques

technique	analyte	multiplex	throughput	ref
Nanoparticle tracking analysis	Proteins (CD9, CD63, CD81)	3	Moderate	(25)
Combined atomic force and fluorescence	Proteins (CD9, CD63, CD81)	3	Moderate	(26)
Automated Raman trapping analysis	Proteins (CD9, CD63, CD81)	3	High	(28)
Flow cytometry	Exosome	2	High	(29)
	Small EVs	no	High	(30)
	Proteins (CD9, CD63, CD81)	3	High	(31)
Localized fluorescence imaging	PD-L1	3	High	(32)
	KRAS^mut^ and/or P53^mu^	2	High	(33)
Plasmonic imaging	EpCAM, HER2, and GPC1	3	High	(34)
Digital droplet PCR	proteins (CD9, CD11b, CD63···*n*)	6	Low	(36)

## EV Diagnostic, Prognostic, and Therapeutic Applications

**Figure 3:**
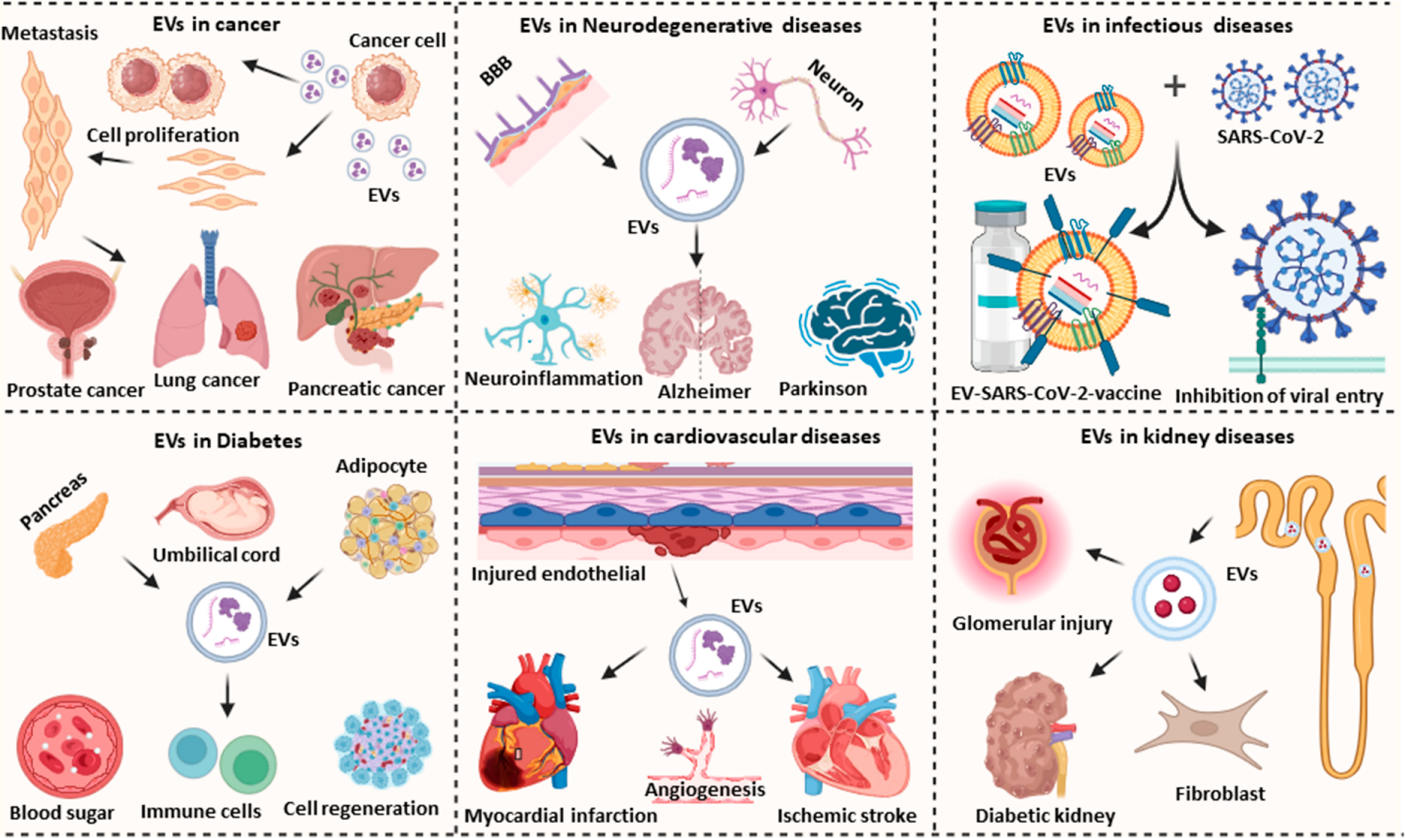
Overview of EV roles in disease and therapeutic applications intended to attenuate cancer-related processes or to decrease tissue injury and enhance tissue repair in neurodegenerative, infectious, diabetes, cardiovascular, and kidney disease. Figure created with Biorender.com.

EVs hold significant promise in diagnostic applications for infectious, chronic, and malignant diseases due to their potential to identify and distinguish specific diseases and discrete disease stages and their response to therapeutic interventions through changes in disease-associated EV biomarkers released by diseased cells (Figure 3). Recent progress in EV isolation and characterization are expected to improve EV diagnostic and therapeutic methods under development and their chance for clinical translation. Notably, machine learning approaches may have value when applied to distinguish and characterize specific EV populations that are necessary for both applications.

### EV Applications for Cancer

Tumor-derived EVs (tEVs) are present at high abundance in the circulation, and thus, tEV diagnostics require much smaller sample volumes than approaches that attempt to detect circulating tumor cells and tumor-derived DNA. EV diagnostics can also provide more reproducible results than these methods since tEV-associated factors are protected from circulating hydrolase activities, while circulating tumor cells can undergo rapid phenotype changes and apoptosis, and tumor-derived DNA is susceptible to nuclease activities. Notably, tEVs can transfer factors that influence tumor development and metastasis, and thus their analysis can provide direct insight into real-time regulation of these processes. EV biomarkers (Table 3) have therefore been evaluated as a promising means to diagnose specific cancers and evaluate their treatment responses, including those associated with pancreatic cancer, ovarian carcinoma, bladder cancer, lung cancer, breast cancer, and colorectal cancer.

**Table 3:** Recent EV Biomarkers Associated with Various Cancer Types^a^

caner type	source	biomarker category	biomarkers	detection methods	application	ref
Pancreatic	Serum	Protein	GPC1, EphA2	nanoparticle based assay	Diagnosis	(37)
Ovarian	Serum	miRNA	miR-1246, miR-141-3p, miR-200a-3p, miR-200b-3p, miR-200c-3p, miR-203a-3p, miR-429	qRT-PCR	Diagnosis, Prognosis	(38)
Bladder	urine	Protein	EphA2	enzyme-linked immunosorbent assay (ELISA)	Diagnosis	(40)
Breast	Plasma	Protein	CA 15-3, CA 125, CEA, HER2, EGFR, PSMA, EpCAM, VEGF	thermophoretic	Diagnosis, prognosis	(41)
Nonsmall cell lung	Plasma	miRNA	miR-323a-3p, miR-325, miR-520-3p, miR-375, miR-3144-5p, miR-1468-3p, miR-3679-5p, miR-4433b-3p, miR-5189-5p, miR-6513-5p	qRT-PCR	Diagnosis	(42)

a Glypican-1: GPC1, ephrin type-A receptor2: EphA2, carcinoma antigen: CA, carcinoembryonic antigen: CEA, human epidermal growth factor receptor 2: HER2, epidermal growth factor receptor: EGFR, prostate-specific membrane antigen: PSMA, epithelial cell adhesion molecule: EpCAM, vascular endothelial growth factor: VEGF.

Early cancer detection and prompt treatment initiation significantly reduces mortality. Pancreatic cancer (PC) represents a strong example of the utility of early diagnosis since most PC is detected at later stages when it is no longer possible to resect the tumor, which is a major contributor to the high mortality of this disease. Appropriate biomarkers are urgently needed for early diagnosis to improve patient outcomes. One recent study suggests that the corecognition of two protein biomarkers (glypican-1 and ephrin type-A receptor 2) on PC tEVs can sensitively detect stage I and II PC cases to increase the potential for surgical intervention required to improve PC patient outcomes.^37^ Similarly, a seven-miRNA serum EV signature identified by miRNA transcriptomics has been reported to distinguish malignant and benign ovarian tissue biopsies with robust performance, predict pre- and postoperative survival, and outperform carbohydrate antigen 125, a current biomarker for detection of stage I epithelial ovarian carcinoma cases (100% vs 79% sensitivity and 80% vs 60% specificity) using an estimate from an initial study.^38^ RNA sequencing has been used to analyze small UEVs to identify pathway changes that could serve as diagnostic markers for kidney cancer.^39^ Finally, a UEV protein biomarker identified by shotgun proteomics (ephrin type-A receptor 2) has been reported to have good diagnostic performance for noninvasive clinical diagnosis of bladder cancer.^40^

Metastasis is a major source of cancer mortality since once a tumor has escaped from its primary tumor site and can no longer be surgically resected, it is difficult to achieve remission, although recent results suggest that diagnostics can permit early detection to allow effective interventions and eliminate metastasis development, while targeted EV therapeutics hold promise for metastatic tumor treatment. For example, one study employed thermophoretic aptamer-based sensors to profile a set of eight breast cancer (BC)-associated EV proteins that did not exhibit crosstalk with soluble proteins to distinguish individuals who had metastatic BC, nonmetastatic BC, and did not have BC, as a means to predict therapeutic response and personalize treatment.^41^ Finally, accurate identification of patients who will not respond to a treatment time is critical to prevent delays in starting an effective treatment regimen that can improve a patient’s treatment outcome. One study has analyzed the miRNA profile of EVs derived from nonsmall cell LC patients to identify an EV miRNA signature to distinguish individuals who did and did not have osimertinib-resistant and -sensitive tumors and thus would and would not respond to this treatment.^42^

Relatively few studies have employed EVs as therapeutics for the treatment of metastatic cancer, but one study has reported that EVs loaded with the antitumor agent oxaliplatin and small interfering RNA against a protein overexpressed in metastatic and oxaliplatin-resistant colorectal cancer cells induced tumor regression and increased survival times in a mouse model of chemoresistant colorectal cancer liver metastases.^43^

### EV Applications for Neurodegenerative Diseases

EVs secreted by central nervous system (CNS) tissue of individuals with neurodegenerative disorders (NDs) can carry factors that regulate these processes and accumulate in the cerebral spinal fluid, where they can serve as biomarkers of disease development and progression. However, there is resistance to and risk associated with obtaining these specimens that reduces their utility. EVs also cross the blood-brain barrier to enter the circulation, however, making it possible to evaluate these biomarkers in a more accessible specimen. One study therefore captured CNS-derived EVs (neural cell adhesion molecule (NCAM)-positive) from plasma samples of patients with subjective cognitive decline, mild cognitive impairment, vascular dementia, or Alzheimer’s disease (AD) and healthy subjects. Analysis of NCAM- and ATP-binding cassette reporter A1 (ABCA1)-positive plasma EVs from these groups with a panel of ND-associated biomarkers (miR-384, neurofilament light chain, and select amyloid β and Tau variants) found that these EVs could distinguish all but the vascular dementia group from the healthy controls and differentiate most of the remaining ND groups from each other.^44^ Similarly, other studies have reported that miR-29c-3p expression in NCAM/amphiphysin-1 dual-labeled plasma EVs is a good predictor of subjective cognitive decline (AUC = 0.789) and AD (AUC = 0.927) and that the levels of certain mitochondrial proteins in neuron-derived plasma EVs can diagnose AD and predict progression of mild cognitive impairment to AD in patients with type 2 diabetes mellitus.^45,46^ Parkinson’s disease (PD), the second most common ND, is linked to alpha-synuclein (α-syn) aggregation, and a recent study has reported that analysis of α-syn and factors associated with impaired insulin signaling and neurodegenerative pathology in neuron-derived plasma EVs could serve as a biomarker of PD.^47^

Notably, EVs can also cross the blood-brain barrier to deliver functionally active cargos that directly reduce inflammation and enhance tissue repair in neural tissue or induce signaling pathways that can promote these processes. It has thus been proposed that EVs could serve as therapeutic delivery systems for the treatment of several major NDs, including AD, PD, ischemic stroke (IS), and others. For example, EVs derived from embryonic stem cells can modulate neuroinflammation and immune responses after an IS via their actions to increase regulatory T cell abundance.^48^ Similarly, EVs derived from pluripotent stem cells can activate endothelial nitric oxide synthase and sirtuin-1 expression in senescent endothelial cells of the blood-brain barrier to rejuvenate them and restore barrier function to protect against vascular leakage leading to IS.^49^ However, while EVs show strong promise for the diagnosis and treatment of certain NDs, clinical studies are still required to validate their diagnostic utility and therapeutic potential.

### EV Applications for Infectious Diseases

EVs are essential in the response to viral and bacterial infections, since EVs secreted by infected cells can often regulate the infection’s immune response or serve as biomarkers of infection, and in certain cases, they can transfer genomic material directly to a recipient cell to cause infection. For example, EVs secreted by severe acute respiratory syndrome coronavirus 2 (SARS-CoV-2)-infected cells strongly express its nucleocapsid protein to promote a pro-inflammatory response and can transfer its RNA genome to infect EV recipient cells.^50^ Conversely, EVs secreted by unmodified red blood cells can attenuate the transmission of SARS-CoV-2 to other cells by competing for phosphatidyl serine receptors employed to promote virus entry, and the antiviral effects of these EVs can be enhanced by loading them with antisense oligonucleotides that target key SARS-CoV-2 genes.^51^

Lipid-based nanoparticles (LNP) have recently been employed as mRNA delivery vehicles for vaccines against SARS-CoV-2 and other infectious diseases, but EV-based vaccine approaches are also being explored due to the robust safety profile of EVs versus LNPs, particularly when there is a need to administer multiple doses over an extended time frame. For example, a recent study evaluated the ability small EVs derived from bovine milk to serve as an oral delivery system for an mRNA vaccine that uses the SARS-CoV-2 receptor-binding domain as its immunogen.^52^ In another study, an engineered EV vaccine platform has proven to be effective in inducing strong immune responses against a Delta variant and two Omicron variants (BA.1 and BA.5) of SARS-CoV-2, to provide better prevention than previous mRNA strategies.^53^ More detailed studies are needed in order to understand the biodistribution and persistence of EV-based mRNA vaccines specific to SARS-CoV-2 and other pathogens.

Bacterial pathogens can also regulate pro-inflammatory and immune responses during infection through the activity of bioactive molecules, including virulence factors and toxins, which are packaged into membrane vesicles (MVs) secreted by these bacteria or incorporated into EVs secreted by cells that serve as their hosts or that phagocytize them. For example, *Mycobacterium tuberculosis* is an intracellular pathogen, and EVs released by their host phagocytes can carry pathogen-derived factors that allow these EVs to serve as circulating biomarkers of infection or to regulate the immune response to infection.^54^

### EV Therapeutics for Diabetes

Recent studies have also explored the therapeutic potential of EVs has been explored both type 1 and type 2 diabetes mellitus (T1DM and T2DM) since EVs regulate immune responses involved in both these disease process. T1DM is an autoimmune condition that results in the loss of pancreatic β cells and a corresponding loss of insulin secretion, leading to a failure or regulated glucose uptake and high systemic glucose levels. T1DM can be managed by insulin injection, but immunotherapy and cell replacement approaches are also under investigation for their potential to attenuate or reverse β cell losses and preserve or restore glucose control. Notably, EVs play important roles in β cell injury and immune responses leading to T1DM and are thus of significant interest for such studies. β cell uptake of EVs secreted by immune cells can induce β cell dysfunction and apoptosis.^55^ Conversely, stem cell-derived EVs have immunomodulatory effects on multiple cells involved in the autoimmune response leading to T1DM,^56^ and treatment with MSC-derived EVs has been reported to stimulate islet β cell regeneration and insulin production to control systemic glucose levels in a rat model of T1DM.^57^ T2DM differs from T1DM as it is primarily caused by dysregulation of the insulin signaling pathway in response to proinflammatory responses that develop during excess weight gain from excess caloric intake. EVs can directly or indirectly influence insulin resistance by promoting these proinflammatory responses, or dysregulating insulin signaling and down-regulating glucose transporter expression.^58^ Conversely, treatment with MSC-derived EVs can increase glucose tolerance in the T2DM animal model,^59^ and recent evidence suggests that adipocyte-derived EVs secreted during excess weight gain can promote glucose secreted insulin secretion to attenuate the development of insulin resistance.^60^ Thus, EV therapeutics may have the potential to attenuate or reverse the underlying mechanisms responsible for T1DM and T1DM.

### EV Therapeutics for Cardiovascular Diseases

EV therapeutics have strong potential as cell-free substitutes for proposed cell-based therapies that are designed to employ stem or progenitor cells to repair disease or injured tissue but which are limited by safety and efficacy concerns. For example, EVs secreted by stem or progenitor cells can attenuate cardiac fibrosis and cardiomyocyte hypertrophy and enhance angiogenesis and thus have significant potential to attenuate pathologies associated with multiple cardiovascular diseases. For example, in depth proteomic investigation reveals that cardiac progenitor cell (CPC)-derived EVs promote *in vitro* angiogenesis and CPC-mediated effects on endothelial cell activation.^61^ Moderate improvement of cardiac function has also been achieved by systemic administration of EVs in several ischemic heart failure models, and EV treatment has been employed in preclinical models of nonischemic heart failure (NIHF).^62^ Most EV treatments for NIHF primarily employ intravenous or intramyocardial administration methods and can achieve a 1.3-fold increase in left ventricular ejection fraction and 3-fold decreases in myocardial infarct size nontreated controls. However, the clinical potential of these methods has not yet been explored due to a limited understanding of their *in vivo* trafficking, internalization, and potential side effects. Engineered EVs are particularly attractive since they can be customized to have greater bioactivity, stability, and cell or tissue targeting ability than naturally occurring EV populations, and multiple studies have evaluated the effectiveness of using engineered EVs to deliver therapeutic cargoes to injured heart tissue using various approaches. For example, one straightforward approach used electroporation to load EVs derived from cardiac progenitor cells with miR-126 to reduce cardiac infarct size, fibrosis, and hypertrophy and improve angiogenesis in a rat model of ischemia reperfusion injury.^63^ EVs can also be engineered to contain specific factors by genetic modulation of their parental cells in order to boost their cardioprotective effects and avoid rapid phagocytosis and clearance. However, several strategies have employed biomaterials (e.g., hydrogels or 3D tissue matrixes) to modulate the behavior of natural EVs, or used direct manipulations to generate hybrid EVs with altered functionality, for use in therapeutics intended for cardiac repair and regeneration.^64^ Such EV-based therapeutics can produce significant improvements in cardiac function, but further studies are needed to optimize their administration routes and dosage regimens and to validate their safety and efficacy.

### EV Therapeutics for Kidney Disease

EVs play a pivotal role in the progression of acute and chronic kidney disease, as UEVs can mediate intranephron communication, including crosstalk between glomerular cells, between glomerular and tubular cells, among cells in various regions of tubules, to modulate the fate of these cells. EVs released by injured kidney cells are associated with increased tubular inflammation and fibrosis leading to kidney dysfunction, and these UEVs carry biomarkers for kidney diagnostics for clinical translation approaches.^65^ EVs secreted by unmodified mesenchymal stem cells (MSCs) and endothelial progenitor cells (EPCs) have been reported to promote the repair of injured kidney tissue in multiple studies. MSC EVs have also been evaluated as therapeutics for acute kidney injury and chronic kidney disease, since these EVs can reduce apoptosis and increase proliferation of tubular cells to improve kidney function after acute kidney injury, reduce renal inflammation, and improve renal oxygenation and function during chronic kidney disease. One notable study also employed a functionalized composite scaffold to retain engineered MSC EVs at a kidney injury site to promote tissue regeneration via synergistic actions of the bioactive materials conjugated to this scaffold and supplied by the engineered EVs.^66^ Similarly, several groups have reported that EPC-derived EVs also promote kidney repair processes. For example, one studied reported that treatment of an anti-Thy1.1-induced rat model of glomerulonephritis by intravenous injection of EPC-derived EVs had a renal-protective effect by inhibiting mesangial cell activation, leukocyte infiltration, and apoptosis, resulting in an overall improvement in renal function.^67^ Another group using an sepsis-induced acute kidney injury found that treatment with EPC-derived EVs could attenuate the renal cell injury response *in vitro* and *in vivo* through a microRNA-93-5p dependent mechanism.^68^ However, despite beneficial effects observed in preclinical studies of acute and chronic kidney disease, clinical investigations are still required for safety, efficacy, and optimal doses.

### Role of EVs as Therapeutic Nanocarriers

Traditional small-molecule drug delivery systems (e.g., synthetic polymer structures, liposomes, and micelles) have properties that limit their utility, including solubility, drug capacity, and biodistribution issues, weak or nonspecific accumulation at a targeted disease site, limited drug capacity, and toxicity effects that can constrain delivery of effective drug doses. Like liposome-based drug delivery systems, small EVs (30–150 nm) can carry hydrophilic and hydrophobic drugs in their aqueous centers and lipid bilayers at relatively high loading capacities. The size, shape, and physical characteristics of EVs can overlap those of some liposome preparations but have a more restricted size range and a much more complex lipid bilayer composition, which includes a diverse array of lipids, proteins, and glycoconjugates. These lipid bilayer features contribute to the high bioavailability and low immunogenicity and toxicity of these vesicles when compared to liposomes.^69^ Specific small EVs employed for drug delivery can be selected based on their native affinity for a target tissue or cell type, or this specificity can be generated by engineering their parental cells to express an affinity receptor specific for a desired surface marker expressed on these tissues or cell types, whereas liposomes have shown substantially lower targeting efficacy.^70^ EVs and liposomes can have comparable half-lives, but this half-life can be extended for engineered small EVs.^71^ Further, small EVs are inherently biocompatible and readily penetrate most tissues, including CNS tissue, unlike liposomes.^72^ EV preparations can be more expensive to generate than liposomes, depending upon the liposome modifications, since EV therapeutics require that select cell lines be cultured at scale under specific growth conditions and that the resulting EV fractions be assessed for their adherence to safety and efficacy criteria. This cost differential can be offset by the superior therapeutic properties of EV, however, particularly when significant differences in the bioavailability, efficacy, or side effect profiles of these two approaches favor the use of EV therapeutics. Modification of EVs for drug delivery thus usually entails either surface ligand modifications produced by direct manipulations, genetic engineering of their parental cells, or careful adjustment of the drug loading procedure to improve loading capacity and EV stability, although other approaches have been employed by some groups. For example, one study employed sonication to encapsulate drug loaded micelles with an EV membrane coating that incorporates a CpG label that binds free transferrin to prolong the circulation time of these EVs and enhance their passage through the blood-brain barrier and targeting to glioblastoma cells through specific recognition by abundant transferrin receptor expression at these sites.^73^ Some groups have also employed methods to increase the duration of EV-based drug-delivery at a target site by incorporating EVs into a matrix, as was done by a group that employed streptavidin to capture biotin-labeled and drug-loaded EVs in a biotin-labeled matrix prior to applying this matrix to a tissue injury site.^74^ Further studies should be performed to improve drug loading procedures for EVs since current drug loading methods (e.g., electroporation, sonication, extrusion, fusion, etc.) can perturb the membrane and *in vivo* properties of the loaded EVs. Passive loading methods are useful for only a subset of drugs, although a recent study suggests that graphene quantum dots that match the chirality of the EV bilayer could permit drug-agnostic loading of hydrophilic and hydrophobic small molecule and biologic drugs at high efficiency.^75^ MSC-derived EVs are also frequently used in drug delivery applications^76^ due to their history of safe use, but significant care must be taken to maintain them in their undifferentiated state. Substantial effort has thus been focused on developing stable cell lines suitable for use in EV drug delivery applications. However, regardless of the EV source, modification status, or modification approach, all EV therapeutics must be carefully characterized to ensure that they exhibit acceptable and consistent biodistribution, toxicity, and immunogenicity.

## Outlook and Challenges

Significant effort has focused on the development of an array of methods that frequently use advanced nanomaterial technologies to permit rapid and sensitive detection of target EV biomarker signatures that exhibit potential for translation in diagnostic or prognostic clinical applications or for the characterization of EV therapeutics. By 2026, it is anticipated that the potential markets for diagnostic and therapeutic electric vehicle applications will have grown from $57.1 and $33.1 million (2021) to $321.9 and $169.2 million.^77^

### Integration of Omics Data from EV specimens

Specific EV biomarkers or biomarker signatures can reflect pathological states present in a target cell or tissue type to improve diagnosis of chronic, malignant, and malignant diseases that may be challenging to identify by current methods. However, to improve patient response to treatment and enable more precise diagnosis, the use of -omics approaches to analyze EV specimens could reveal specific information about potential disease-associated changes in the EV lipid, protein, and nucleic acid components to provide more accurate diagnoses and better monitor patient responses to treatment. Notably, the use of EV transcriptomics, proteomics, glycomics, and lipidomics data has facilitated the identification of specific biomarkers for neurodegenerative disorders, cardiovascular and autoimmune diseases, and several cancers, although such analyses require significant bioinformatic expertise particularly when integrating data produced by different -omics approaches.

### Clinical Translation

Nanotechnology and microfluidic advances and the improved understanding of the characteristics of EVs derived from cells with distinct disease phenotypes have fueled the development and clinical acceptance of EV-based diagnostic applications. The best example of this acceptance to date is the recent authorization by the United States Food and Drug Administration of an assay that quantifies three UEV RNAs to diagnose prostate cancer.^78^ Clinical trials have been established for more than 50 EV-based assays (www.clinicaltrials.gov), but only a few of these have qualified at all stages to date, and successful completion of these validation studies represents a substantial bottleneck to the adoption of EV-based clinical applications. Successful completion of these studies can also be compromised by the design of the initial biomarker discovery and validation studies if these studies do not contain appropriate disease and control populations, have sufficient specimens in each category to allow robust statistical analyses, include appropriate positive and negative controls, or produce results that achieve sufficient analytical sensitivity, precision, or consistency.

EV therapeutics have distinct issues that can influence their adoption in clinical applications. Several EV properties render them useful as native or engineered therapeutics, but all EV therapeutics must be characterized to confirm their consistency, which can be affected by their production, modification, and purification procedures. EV therapeutics, like all drugs, also require extensive validation studies to confirm their safety and efficacy, and most candidate EV therapies have yet to enter this phase. Early development studies should also consider the production, handling, transportation, and storage parameters, as these may limit the activity or utility of the final product regardless of its effectiveness in research and validation studies.

### Personalized Medicine

EVs hold great promise for personalized medicine, as they can contain a wealth of information about the phenotype of diseased cell populations and can thus permit the design of therapeutic regimens tailored to individual cases. Notably, EV molecular signatures associated with cancer can provide essential information about key mutations and cell and microenvironment phenotypes to predict disease progression and the response to specific interventions, and thus allow the development of tailored treatment plans based on a patient risk’s profiles and tumor phenotype. Tumor heterogeneity can complicate such approaches and requires the use of omics approaches that can capture the full range and relative contributions of cancer subtypes. Similar approaches may also be useful for chronic infectious diseases to evaluate the risk or disease progression and response to specific treatments.

## Conclusions

Existing approaches for EV capture, detection, and analysis have limitations that make them difficult to employ in clinical applications. Nevertheless, the wealth of phenotypic information contained by EVs has made assays evaluating EV biomarkers an attractive means to diagnose disease, predict its development and progression, and evaluate its response to treatment, as demonstrated by the growing number of clinical trials for EV diagnostics. Similarly, EV-based therapeutics exhibit several properties that make them attractive candidates for the targeted delivery of biologic and small molecule cargoes. EV isolation and analysis approaches that integrate microfluidics with highly sensitive detectors (nanotechnology, advanced optics, digital readouts, etc.) should improve the sensitivity of EV diagnostics to increase their potential for adoption in clinical applications, while recent approaches used to develop EV-based therapeutics are likely to improve drug bioavailability and reduce side effects due to their potential to selectively target drug delivery to specific cells or tissues. The domain for single EV analysis techniques, which are now under rapid development, also have potential to analyze EVs from targeted cell populations to understand specific mechanisms active in a heterogeneous disease state and allow personalized treatments for improved outcomes. Several EV applications have reached early stage clinical trials, but the progress of EV research would benefit from the adoption of standard EV isolation and characterization procedures, EV controls, and data analysis and reporting methods and development of improved guidance for EV-specific regulatory approval procedures.

## VOCABULARY

Extracellular vesicles: Extracellular vesicles are nanosized membrane-enclosed vesicles released by all cells that carry an array of biomolecules.
Nanomaterials: Nanomaterials are structures that range from 1 to 100 nm in at least one dimension and which have special properties due to their small size.
Quantum dots: Quantum dots are nanomaterials <10 nm in diameter that have unique optical and electronic properties as a result of their quantum mechanical effects.
Microfluidics: Microfluidics refers to systems that contain networks of small channels (10–100 μm in width) that are used to process small fluid volumes.
Metastasis: Metastasis refers to the spread of cancer cells from the initial tumor site to distant sites.
